# Online patient simulation training to improve clinical reasoning: a feasibility randomised controlled trial

**DOI:** 10.1186/s12909-020-02168-4

**Published:** 2020-07-31

**Authors:** Ruth Plackett, Angelos P. Kassianos, Maria Kambouri, Natasha Kay, Sophie Mylan, Jenny Hopwood, Patricia Schartau, Shani Gray, Jessica Timmis, Sarah Bennett, Chris Valerio, Veena Rodrigues, Emily Player, Willie Hamilton, Rosalind Raine, Stephen Duffy, Jessica Sheringham

**Affiliations:** 1 Department of Applied Health Research, University College London, 1-19 Torrington Place, London, WC1E 7HB UK; 2 Institute of Education, UCL, London, UK; 3 Department of Global Health and Development, London School of Hygiene and Tropical Medicine, London, UK; 4 UCL Medical School, UCL, London, UK; 5 Primary Care and Population Health Department, UCL, London, UK; 6 Norwich Medical School, UEA, Norwich, UK; 7 Institute of Health Service Research, University of Exeter, Exeter, UK; 8 Wolfson Institute of Preventive Medicine, QMUL, London, UK

**Keywords:** Clinical reasoning, Simulation, Virtual patient, Online learning, Medical students, Medical education, Feasibility study

## Abstract

**Background:**

Online patient simulations (OPS) are a novel method for teaching clinical reasoning skills to students and could contribute to reducing diagnostic errors. However, little is known about how best to implement and evaluate OPS in medical curricula. The aim of this study was to assess the feasibility, acceptability and potential effects of eCREST — the electronic Clinical Reasoning Educational Simulation Tool.

**Methods:**

A feasibility randomised controlled trial was conducted with final year undergraduate students from three UK medical schools in academic year 2016/2017 (cohort one) and 2017/2018 (cohort two). Student volunteers were recruited in cohort one via email and on teaching days, and in cohort two eCREST was also integrated into a relevant module in the curriculum. The intervention group received three patient cases and the control group received teaching as usual; allocation ratio was 1:1. Researchers were blind to allocation. Clinical reasoning skills were measured using a survey after 1 week and a patient case after 1 month.

**Results:**

Across schools, 264 students participated (18.2% of all eligible). Cohort two had greater uptake (183/833, 22%) than cohort one (81/621, 13%). After 1 week, 99/137 (72%) of the intervention and 86/127 (68%) of the control group remained in the study. eCREST improved students’ ability to gather essential information from patients over controls (OR = 1.4; 95% CI 1.1–1.7, *n* = 148). Of the intervention group, most (80/98, 82%) agreed eCREST helped them to learn clinical reasoning skills.

**Conclusions:**

eCREST was highly acceptable and improved data gathering skills that could reduce diagnostic errors. Uptake was low but improved when integrated into course delivery. A summative trial is needed to estimate effectiveness.

## Background

Clinical reasoning — the thought processes used by clinicians during consultations to formulate appropriate questions — is essential for timely diagnosis of disease [1–4]. Providing training in clinical reasoning as early as possible in medical education could improve reasoning skills in future doctors, as it provides a scaffold for future learning, and retraining reasoning can be challenging [5, 6]. However, in undergraduate medical education, there is a lack of explicit teaching on clinical reasoning and the development and delivery of additional high quality and consistent clinical reasoning teaching potentially increases burden on faculty’s already stretched time and resources [2, 7, 8].

Online patient simulations (OPS) are a specific type of computer-based program that simulates real-life clinical scenarios and could support teaching reasoning skills [7, 9]. Theories of cognition suggest that exposure to a large number of different clinical cases via simulations could improve reasoning by restructuring and building more complex mental representations [10, 11]. Learning by experience also facilitates reflection, which helps students to retain skills [12]. OPS can be blended with traditional teaching and offers the opportunity for students to practise data gathering and make diagnoses without burdening patients [9, 13]. OPS also have pragmatic benefits; once developed they are lower in cost to deliver, can be distributed widely, completed remotely, tailored to the learner and frequently updated [9, 14]. Nevertheless, using technology-enhanced learning (TEL) may have its own limitations, such as the potential for lack of engagement from users and faculty, lack of fidelity with real patient consultations and limited TEL skills of faculty [15, 16].

There is currently little empirical evidence to support the use of OPS for assisting clinical reasoning skills teaching. The few studies conducted were not methodologically robust and difficult to interpret due to the poor validity of clinical reasoning outcome measures [17–20]. Furthermore, most previous studies had limited information on the feasibility of introducing a novel tool into a curriculum and evaluating them using a robust research method, such as a randomised controlled trial (RCT) [17, 18]. Understanding the feasibility of testing OPS using an RCT study design is necessary before a summative RCT can estimate effectiveness [21, 22].

This research aimed to inform the design of a summative evaluation of an OPS to support teaching of reasoning skills in medical schools. The development of this OPS, the Electronic Clinical Reasoning Educational Simulation Tool (eCREST), is reported elsewhere [23]. Briefly, eCREST shows three videos of patients (played by actors) presenting to their primary care physician (PCP) with respiratory problems that could be indicative of serious conditions like lung cancer. The student gathers information from the patient, while continually being prompted to review their differential diagnosis. After each case they are asked to make a final differential diagnosis and receive feedback. Patient cases were developed with a small group of real patients who co-wrote the scripts of the vignettes and helped to identify pertinent clinical and behavioural characteristics for the simulated cases [24].

This study sought to obtain evidence as to the feasibility of a trial through:

1. identifying optimal recruitment strategies, measured by student uptake;
2. testing the acceptability to students via student retention and feedback;
3. testing the validity and measuring the possible effect sizes of two clinical reasoning outcome measures.

## Methods

### Study design and participants

A multicentre parallel feasibility RCT was conducted across three UK medical schools: A, B and C. We followed the CONSORT statement for reporting pilot or feasibility trials [25]. Eligible participants were final year undergraduate medical students. The curricula of the medical schools varied. Schools A and B implemented a traditional integrated/systems-based curriculum. School C followed a problem-based learning (PBL) curriculum. Ethical approval was gained from participating medical schools. Participants were recruited from March 2017–February 2018 in two cohorts. Cohort one was recruited after final examinations in April–July 2017, through advertisements in faculty newsletters and lecture ‘shout outs’. Cohort two was recruited prior to final examinations in October 2017–February 2018. School C students were only recruited in cohort two. Cohort two were invited to participate through the faculty online learning management platforms (e.g. Moodle), advertisements on social media, faculty newsletters, and lecture ‘shout outs’. As this was a feasibility trial, a sample size calculation was not required.

### Outcomes

#### Feasibility and acceptability

Feasibility was measured by assessing student uptake by school and cohort. Acceptability was measured by retention rates and a survey adapted from previous studies, consisting of six statements on the perceptions of eCREST [26, 27].

#### Clinical reasoning outcome measures

Clinical reasoning was measured using the Flexibility in Thinking (FIT) scale of the Diagnostic Thinking Inventory (DTI), which is a self-reported measure [28]. The FIT (21 items) measures thought processes used in the diagnostic process, including the ability to generate new ideas, understand alternative outcomes and self-reflect. Higher scores on the FIT sub-scale are indicative of better clinical reasoning skills. The sub-scale has demonstrated validity to detect differences between student and professional reasoning. The internal consistency and test re-test reliability were acceptable [28, 29].

Clinical reasoning was also measured using an observed measure of clinical reasoning by using data from an additional eCREST patient case that students received 1 month after baseline. This measure comprised indicators of three cognitive biases that eCREST sought to influence: the unpacking principle, confirmation bias and anchoring. These were identified by previous clinical reasoning research [24, 30, 31]. The unpacking principle refers to the tendency to not elicit the necessary information to make an informed judgement. Confirmation bias is when a clinician only seeks information to confirm their hypothesis. Anchoring occurs when clinicians stick to an initial hypothesis despite contradictory information [32]. eCREST prompts students to reflect throughout a consultation and provides feedback that enables them to reflect on their performance afterwards [33]. By reflecting, students would be more likely to attend to evidence inconsistent with their hypotheses and consider alternatives, thereby reducing the chance of confirmation bias and anchoring. Reflection also encourages students to explore their hypotheses thoroughly, ensuring that they elicit relevant information from patients, reducing the effect of the unpacking principle [33, 34].

The observed measure assessed ‘essential information identified’ by measuring the proportion of essential questions and examinations asked, out of all possible essential examinations and questions identified by experts. This aimed to detect the influence of the unpacking principle on reasoning, as it captured whether the students elicited enough essential information to make an appropriate decision. The ‘relevance of history taking’ was measured by assessing the proportion of all relevant questions and examinations asked, out of the total questions and examinations asked by the student. This aimed to detect susceptibility to confirmation bias by capturing whether they sought relevant information. Finally, it measured ‘flexibility in diagnoses’ by counting the number of times students changed their diagnosis. This reflected how susceptible students were to anchoring, by measuring their willingness to change their initial differential diagnosis. All measures were developed by RP and three clinicians (PS, SG & JT). The content validity of the observed measure of clinical reasoning was tested with two clinicians (SM, JH).

#### Diagnostic choice

Diagnostic choice was captured in the additional patient case. Selection of the most important diagnosis that the student should not have missed was used to assess how well the observed measure of reasoning predicted diagnostic choice.

#### Knowledge

Relevant medical knowledge was measured by 12 single best answer multiple choice questions (MCQs). We hypothesised that greater knowledge is associated with better clinical reasoning skills, consistent with the literature [4, 35]. The MCQs were developed by clinicians (NK, SM, JH & PS) in consultation with other clinicians.

### Procedure

**Fig. 1:**
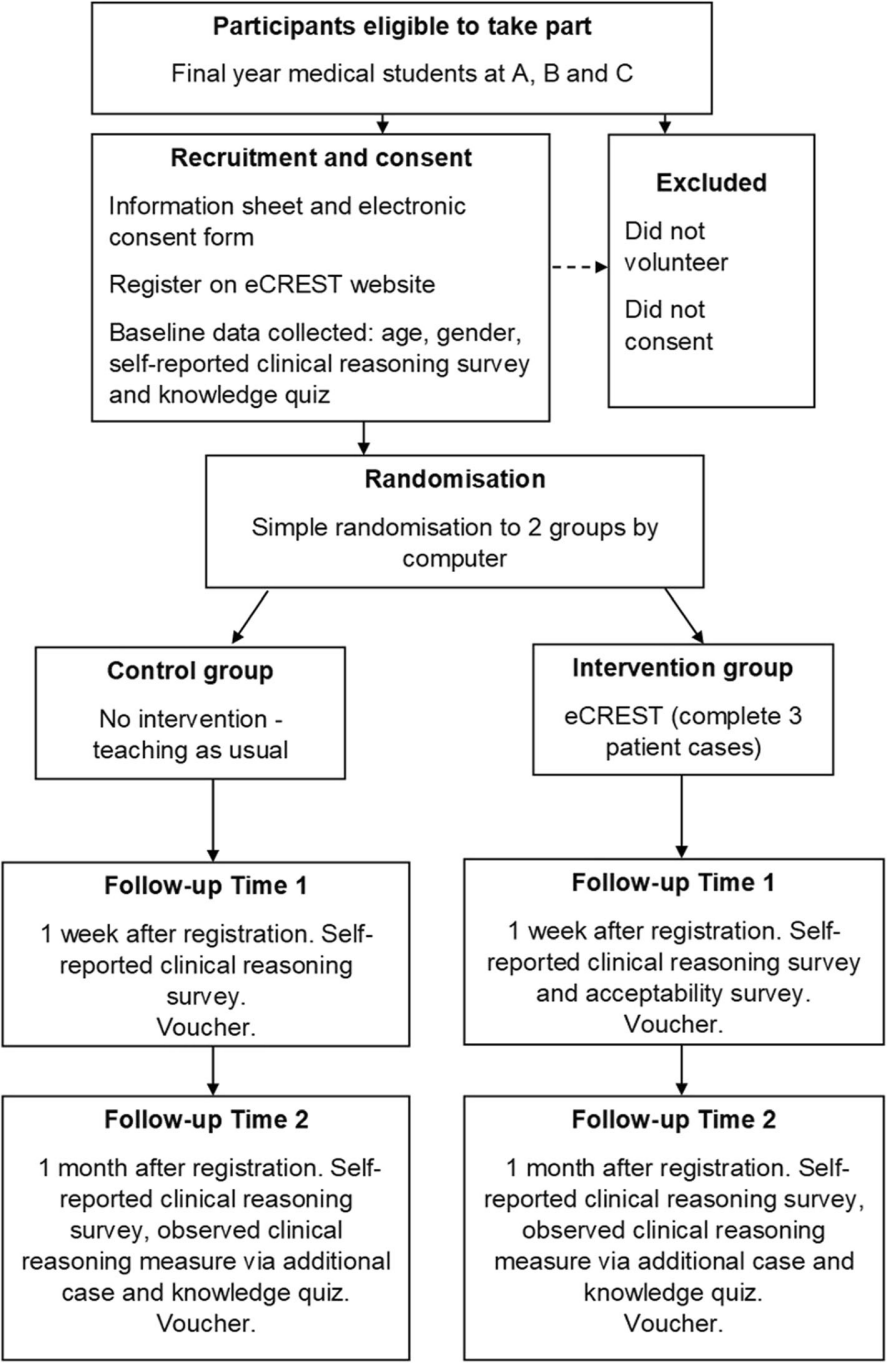
Flow diagram illustrating study procedure

The trial procedure is outlined in Fig. 1, which shows how and when data from participants were collected. To address ethical concerns the information sheet made it clear to students that: participation in the trial was voluntary, they could withdraw at any stage, participation would not impact upon their summative assessments and only anonymised aggregate data would be shared. Students who provided written consent online were allocated to intervention or control groups using simple randomisation. Researchers were blind to allocation, completed by a computer algorithm. Randomisation was not precisely 1:1, as five students were mistakenly automatically allocated to the intervention group. The intervention group received three video patient cases in eCREST, all presenting with respiratory or related symptoms to their primary care physician [23]. The control group received no additional intervention and received teaching as usual. To address concerns that students in the control group may be disadvantaged by not having access to eCREST, we ensured that the control group had access to eCREST at the end of the trial.

### Data analysis

#### Feasibility and acceptability

Uptake was calculated as the percentage of students who registered out of the total number of eligible students. Retention was calculated as the percentage of students who completed T1 and T2 follow-up assessments out of all registered. Acceptability was measured by calculating the percentage of students who agreed with each statement on the acceptability questionnaire. Uptake, retention and acceptability were compared between schools and cohorts using chi-squared tests.

#### Clinical reasoning outcomes

##### Validity and reliability

Internal consistency of the self-reported clinical reasoning measure was assessed using Cronbach’s alpha. Construct validity of the self-reported and observed clinical reasoning measures was assessed by correlating the reasoning and knowledge outcomes, using Spearman’s rank correlation coefficient. To estimate the predictive validity of the clinical reasoning measures, the self-reported measure and observed measure of clinical reasoning were correlated with diagnostic choice. The analyses were undertaken for the aggregated dataset then separately for the intervention and control groups.

##### Effect sizes

Independent t-tests were used to compare mean self-reported clinical reasoning scores between intervention and control groups at T1 and T2. A mixed factorial ANOVA was used to assess change in self-reported clinical reasoning over time, between groups and interaction effects. Logistic regression analyses were conducted to assess the ‘essential information identified’ and the ‘relevance of history taking’. These outcomes were proportional data, so were transformed by calculating the log odds of the outcomes [36, 37]. Group allocation was the only predictor variable in each model, as knowledge did not significantly differ between the groups at baseline. A multinomial logistic analysis was carried out to assess ‘Flexibility in diagnoses’. A complete case analysis was undertaken, such that those students who had missing data were excluded from analysis. Analyses were conducted using Stata Version 15, with *p* ≤ 0.05 considered statistically significant [38].

## Results

### Feasibility and acceptability

**Fig. 2:**
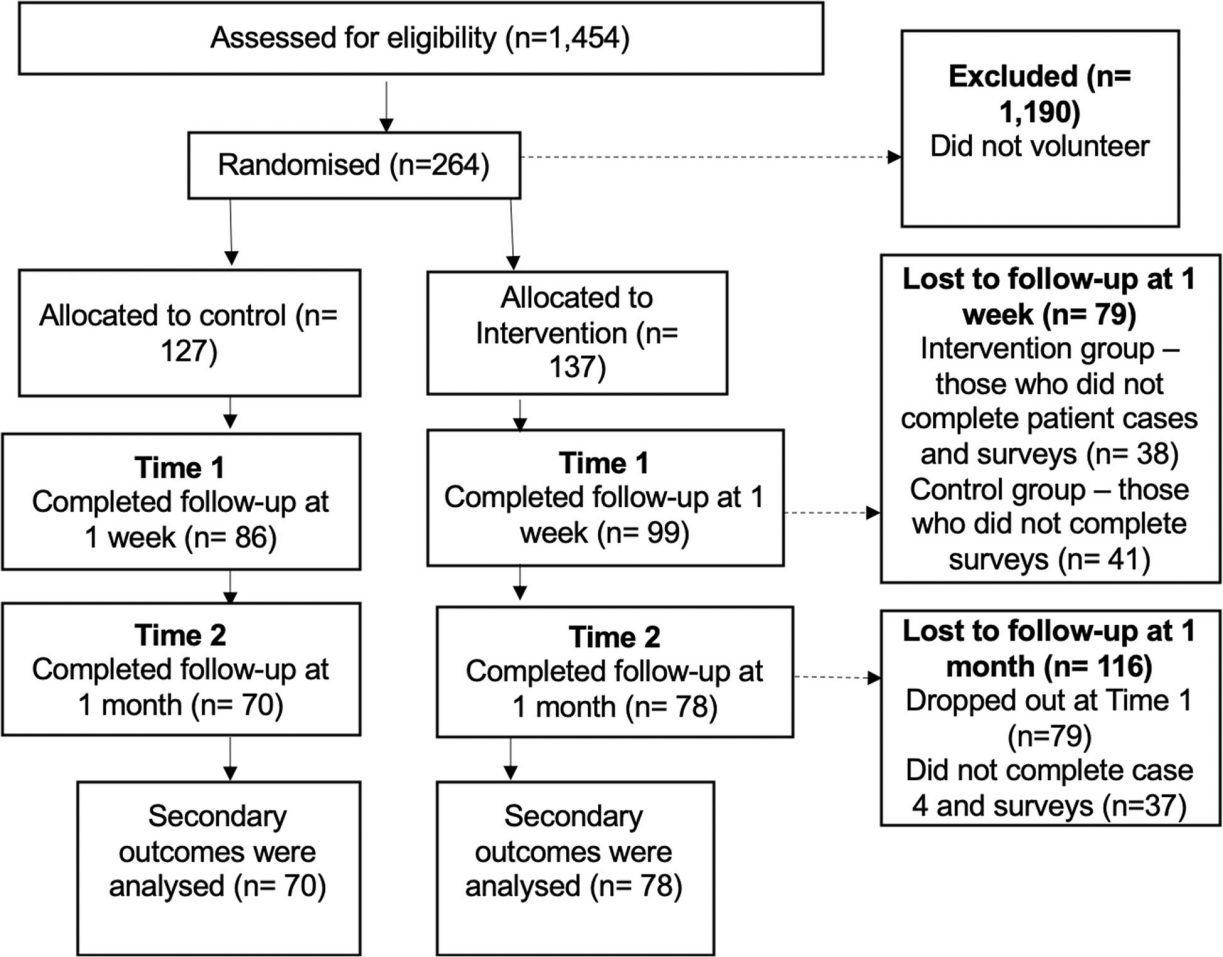
Flow diagram showing how participants progress through the feasibility RCT

Across the three UK medical schools, 264 students participated (18.2% of all eligible, Fig. 2). Recruitment was greater for students in cohort two (*n* = 183/833, 22%) than those in cohort one (*n* = 81/621, 13%). Uptake was slightly greater at school B (*n* = 136/610, 22%), followed by A (*n* = 112/696, 16%) and C (*n* = 16/148, 11%). Uptake was similar at schools A (*n* = 44//336, 13%) and B (*n* = 37/285, 13%) in cohort one. However, uptake was greater at school B (*n* = 99/325, 31%) than A (*n* = 68/360, 19%) and C (*n* = 16/148, 11%) in cohort two. Participant characteristics are shown in Table 1 and no significant differences between the intervention and control group were observed.

**Table 1:** Participant characteristics at baseline

	Intervention group	Control group	***P*** value
Age (Years): *n* (%)			
20–22	4 (2.9)	1 (0.8)	
23–24	73 (53.3)	79 (62.2)	
25–26	39 (28.5)	29 (22.8)	
27–28	11 (8.0)	10 (7.9)	
> 29	10 (7.3)	8 (6.3)	0.49
Total n	137	127	
Gender: *n* (%)			
Female	64 (46.7)	58 (45.7)	
Male	73 (53.3)	69 (54.3)	0.87
Total n	137	127	
Self-reported clinical reasoning skills at baseline: mean (SD)	83.1 (9.6)	83.5 (8.8)	0.75
Total n	122	118	
Knowledge at baseline: mean (SD)	9.2 (1.8)	9.3 (1.6)	0.22
Total n	125	126	

Notes: Baseline n varied for demographics, self-reported clinical reasoning skills and knowledge as some students did not complete all information. Results are presented as number and percentage of students or mean and standard deviation for each group. Comparisons were made using t-tests for means and Chi-squared tests for percentages. *P* Value less than 0.05 was considered significant

There was no significant difference detected in retention between the intervention and control groups 1 week after baseline, 72 and 68% respectively (χ^2^ (1) = 0.65, *p* = 0.42), or after 1 month, 57 and 55% respectively (χ^2^ (1) = 0.34, *p* = 0.56, Fig. 2). There was no significant difference found in the proportion of students at each school who stayed in the study 1 week after baseline. However, there was significantly poorer retention at school A after 1 month (*n* = 47/112, 42%) than at school B (*n* = 83/136, 61%) and C (*n* = 10/16, 63%), χ^2^ (2) = 9.58, *p* = 0.008. Those in cohort one were significantly less likely to stay in the study one-week post baseline (*n* = 45/81, 56%) than those in cohort two (*n* = 140/183, 77%), χ^2^ (1) = 11.75, *p* = 0.001. This was also observed one-month post baseline (*n* = 29/81, 36% and *n* = 111/183, 61% respectively), χ^2^ (1) = 13.92, *p* = 0.000.

Most students (> 80%) agreed that eCREST helped them learn clinical reasoning skills and that they would use it again without incentives (Table 2). There were no significant differences detected between the schools. However, those in cohort two were significantly more likely than cohort one to agree that: eCREST helped to improve their clinical reasoning skills (87.7% vs 64.0%), χ^2^ (2) = 7.5, *n* = 98, *p* = .024); eCREST enhanced their overall learning (93.2% vs 64.0%), χ^2^ (2) = 13.7, *n* = 98, *p* = .001) and that they would use eCREST again without an incentive (97.3% vs 52.0%), χ^2^ (2) = 31.8, *n* = 98, *p* = .000).

**Table 2:** Intervention group medical student responses to the acceptability survey

	Strongly agree/ Agree	Neither agree or disagree	Strongly disagree/ Disagree
**Statement**	***n*(%)**	***n*(%)**	***n*(%)**
It was easy to navigate through eCREST	96/98 (98)	1/98 (1)	1/98 (1)
The level of difficulty of the material was appropriate	95/98 (97)	3/98 (3)	0/98 (0)
eCREST should be used to supplement traditional teaching	88/98 (90)	9/98 (9)	1/98 (1)
eCREST helped me to learn clinical reasoning skills to apply to clinical work	80/98 (82)	15/98 (15)	3/98 (3)
Overall, using eCREST enhanced my learning	84/98 (86)	13/98 (13)	1/98 (1)
I would use eCREST in the future without an incentive	84/98 (86)	10/98 (10)	4/98 (4)

Notes: results are taken from across all 3 schools. 98 students in the intervention group completed the acceptability survey at Time 1

### Clinical reasoning outcomes

#### Validity

The internal consistency of the self-reported clinical reasoning measure was adequate (Cronbach’s α = 0.66). Correlations between self-reported and observed clinical reasoning outcome measures, and knowledge and clinical outcomes are shown in Table 3. There was a mostly positive but non-significant correlation between the self-reported clinical reasoning measure and the observed clinical reasoning measure. The self-reported clinical reasoning measure had a weak but significant positive correlation with knowledge for aggregated data (*r*_*s*_ = 0.13, *p =* 0.037, *n* = 240). The observed clinical reasoning measure was positively but not significantly correlated with knowledge. The self-reported clinical reasoning measure at baseline and the observed clinical reasoning measure were positively but not significantly correlated with identification of the most serious diagnosis.

**Table 3:** Mean scores and correlations between measures of clinical reasoning, knowledge and diagnostic choice

Reasoning measure	Group^**a**^	N	Mean Scores (SD)	Correlation coefficients					
				Self-reported clinical reasoning at baseline^**b**^		Knowledge at baseline^**c**^		Diagnostic choice^**d**^	
**Self-reported clinical reasoning skills**				r_s_^e^	*p*	r_s_	*p*	r_s_	*p*
Baseline	I	122	83.1 (9.6)			0.17	*0.057*	−0.01	*0.927*
	C	118	83.5 (8.8)			0.10	*0.270*	0.09	*0.463*
	All	240	83.3 (9.2)			0.14	0.037*	0.04	*0.663*
Time 1^f^	I	99	84.1 (10.3)						
	C	86	82.4 (9.0)						
	All	185	83.3 (9.8)						
Time 2^g^	I	75	84.4 (9.8)						
	C	65	82.0 (9.4)						
	All	140	83.3 (9.7)						
**Observed clinical reasoning skills**^**h**^									
Essential information identified^i^	I	78	61.6% (17.6)	−0.01	*0.957*	0.09	*0.423*	0.09	*0.435*
	C	70	53.3% (15.8)	0.03	*0.798*	0.22	*0.074*	0.13	*0.303*
	All	148	57.7% (17.2)	0.01	*0.865*	0.15	*0.78*	0.12	*0.144*
Relevance of history taking^j^	I	78	81.4% (10.5)	0.06	*0.578*	0.06	*0.623*	0.12	*0.304*
	C	70	84.6% (10.6)	0.02	*0.893*	0.11	*0.356*	0.08	*0.518*
	All	148	82.9% (10.6)	0.03	*0.704*	0.07	*0.415*	0.10	*0.251*
Flexibility in diagnoses^k^	I	78	3.2 (1.0)	0.01	*0.919*	0.17	*0.137*	0.01	*0.933*
	C	70	3.0 (1.0)	0.09	*0.439*	0.04	*0.772*	0.23	*0.054*
	All	148	3.1 (1.0)	0.05	*0.542*	0.11	*0.186*	0.12	*0.137*

^a^Intervention (I) and Control group (C)

^b^Measured using the Flexibility in Thinking scale (Bordage et al. 1990)

^c^Measured by 12 multiple choice respiratory medicine questions

^d^Measured by whether the most important diagnosis was selected for a patient case

^e^r_s_ denotes Spearman’s rank correlation coefficient

^f^Time 1 = one week after registration

^g^Time 2 = one month registration

^h^Measured by performance on a patient case delivered by eCREST to all students

^i^Percentage of essential information from gathered from patient case out of possible essential information available

^j^Percentage of relevant information from gathered from patient case out of all information student gathered

^k^Number of times changed diagnosis

* indicates *p* ≤ 0.05

#### Effect sizes

The intervention group had non-significantly higher self-reported clinical reasoning skills than the control group at Time 1 (84.1 vs 82.4, *p* = 0.26) and Time 2 (84.4 vs 82.0, *p* = 0.15). There was no significant effect of group allocation, (*F* (1)=0.00, *p* = 0.97, *n* = 136) time, (*F* (2)=0.01, *p* = 0.99, *n* = 136) or interaction between group allocation and time, *F* (2)=0.48, *p* = 0.62, *n* = 136.

Table 4 shows logistic regression analyses comparing observed clinical reasoning skills between the intervention and control groups. The intervention group identified significantly more essential information than the control group (62% vs 53%). The control group sought more relevant information than the intervention group (85% vs 81%) but this difference was not significant. Students in both groups changed their diagnoses at least twice. The intervention group changed their diagnoses more often than controls, but the difference was not statistically significant.

**Table 4:** Logistic regression analyses comparing clinical reasoning skills between intervention and control groups

Observed clinical reasoning skills^a^	Trial group	***n***	Mean (***SD***)	Odds Ratio (95% CI)	*P* value
Essential information identified^b^	Intervention	78	61.6% (17.6)		
	Control	70	53.3% (15.8)		
				1.40 (1.12, 1.75)	0.003*
Relevance of history taking^c^	Intervention	78	81.4% (10.5)		
	Control	70	84.6% (10.6)		
				0.79 (0.62, 1.01)	*0.064*^*d*^
Flexibility in diagnoses^e^	Intervention	78	3.2 (1.0)		
	Control	70	3.0 (1.0)		
			2 (base)		
			3	1.48 (0.68, 3.24)	*0.323*
			4	1.63 (0.68, 3.92)	*0.270*
			5	2.46 (0.55, 11.00)	*0.23*9
			6	1.77 (0.07, 20.76)	*0.887*^*f*^

^a^Measured by students’ performance on a patient case delivered by eCREST

^b^Percentage of essential information from gathered from patient case out of possible essential information available

^c^Percentage of relevant information from gathered from patient case out of all information student gathered

^d^χ^2^ (1) =3.44

^e^Number of times changed diagnosis

^f^χ^2^ (4) =2.24, *p* = 0.692

*indicates *p* ≤ 0.05

## Discussion

This feasibility trial of eCREST demonstrated that optimal recruitment and retention was achieved when the tool was integrated into curricula, as seen in the greater uptake for cohort two than cohort one. eCREST was also highly acceptable to students, suggesting it would be feasible to conduct a summative trial to estimate effectiveness of OPS in medical schools, if there was course integration.

Uptake, retention and acceptability were higher amongst students in cohort two than cohort one. Providing eCREST to students before exams, and advertising and integrating with students’ online learning management platforms, may have made eCREST more accessible and useful for revision. The low uptake at school C compared to the other sites was possibly due to their different curriculum design. Students at school C may have had more exposure to patient cases than A and B, as part of their PBL designed curriculum, reducing the need for simulated cases [39]. In a summative trial, recruitment efforts made for cohort two would likely yield greater uptake but uptake may vary across schools with different curricular approaches. Given the effect sizes observed in this study, we estimate that a sample size of 256 would be sufficient to detect a significant increase in the proportion of essential information identified. However, schools considering implementing OPS should be mindful that the acceptability of OPS to students could be affected by barriers to adoption at faculty level, such as insufficient technological capabilities to adapt and manage OPS and a lack of alignment of OPS content with educators’ needs [40]. To explore factors that might affect uptake we are undertaking qualitative research to understand how students’ reason and interact with eCREST and to understand from a faculty perspective how novel tools like eCREST can be implemented into curricula.

A lack of validated measures to assess clinical reasoning skills has been reported in medical education literature [10, 18]. This study assessed the suitability of two potential measures. The FIT self-reported measure of clinical reasoning had some construct validity but poor predictive validity. It also may not capture actual reasoning as it is a self-reported measure. The observed measure of clinical reasoning developed for this study measured real-time thought processes involved in making decisions but had poor construct and predictive validity. Difficulties in establishing the validity of any clinical reasoning measure arise because of the subjective nature and context-dependency of clinical reasoning [10]. In future, predictive validity of observed measures may be better established by applying rubrics to several patient cases and correlating with measures of summative performance that require strong clinical reasoning, such as objective structured clinical examination (OSCE) performance on related cases.

The observed clinical reasoning outcomes suggest that eCREST could reduce the effects of the unpacking principle, and confirmation and anchoring biases. eCREST helped students to elicit more information from patients on symptoms indicative of serious diseases and encouraged students to challenge their original hypotheses. Nevertheless, it is also possible that eCREST encouraged students to be less efficient when gathering information, by increasing the number of questions they asked. This may be an unfeasible approach in clinical practice given the significant time pressures clinicians face. However, given medical students’ limited level of experience, and exposure to patients and knowledge, this strategy may be appropriate when managing patients with non-specific symptoms in primary care [13].

### Limitations

There was relatively low uptake in the study (18%) but the extensive demands of medical curricula often result in low uptake of additional resources. Uptake was higher in this study than some previous online learning studies that relied on medical student volunteers and the sample size was ample for the purposes of the feasibility RCT [41, 42]. This study demonstrated that some integration of eCREST into the curricula in cohort two was possible and led to greater uptake, acceptability and retention. However, as this study relied on volunteers there was a risk of selection bias. Students who took part might have been different than those who did not.

A further limitation of this study is that it was a complete case analysis, which assumes data were missing at random and those who dropped out were similar to those who remained. It was not possible to follow up those who dropped out to determine whether they had different views of eCREST or different patterns of reasoning. Furthermore, the observed measure of clinical reasoning was only collected at T2 and not at baseline. It is possible that the two groups differed on this measure at baseline. However, no differences in self-reported clinical reasoning were detected between groups at baseline.

## Conclusions

This feasibility RCT has illustrated the importance of integration into the course when evaluating OPS in medical education. It would be feasible to conduct a summative trial to assess the effectiveness of eCREST on medical students’ clinical reasoning skills in multiple medical schools, if it were appropriately positioned in a curriculum to benefit student learning. Further testing of the validity of using OPS as an outcome measure is needed. Nevertheless, this study provides evidence that OPS can be used to support face-to-face teaching to reduce cognitive biases, which may help future doctors in achieving timely diagnoses in primary care.

## Data Availability

The datasets used and/or analysed during the current study are available from the corresponding author on reasonable request.

## Abbreviations

OPS: Online patient simulations
eCREST: Electronic Clinical Reasoning Educational Simulation Tool
TEL: Technology-enhanced learning
PCP: Primary care physician
RCT: Randomised controlled trial
MCQs: Multiple choice questions
FIT: Flexibility in Thinking scale
DTI: Diagnostic Thinking Inventory
OSCE: Objective Structured Clinical Examination
